# Surface Degradation Mechanism on CH_3_NH_3_PbBr_3_ Hybrid Perovskite Single Crystal by a Grazing E-Beam Irradiation

**DOI:** 10.3390/nano10071253

**Published:** 2020-06-28

**Authors:** Heriyanto Syafutra, Jung-Ho Yun, Yuya Yoshie, Miaoqiang Lyu, Sakura Nishino Takeda, Masakazu Nakamura, Lianzhou Wang, Min-Cherl Jung

**Affiliations:** 1 Division of Materials Science, Nara Institute of Science and Technology, Nara 630-0192, Japan; heriyanto.syafutra.hi0@ms.naist.jp (H.S.); yoshie.yuya.ys9@ms.naist.jp (Y.Y.); sakura@ms.naist.jp (S.N.T.); mnakamura@ms.naist.jp (M.N.); 2 Nanomaterials Centre, School of Chemical Engineering and Australian Institute for Bioengineering and Nanotechnology (AIBN), University of Queensland, QLD 4072, Australia; m.lyu@uq.edu.au; 3 Division of Materials Science, Faculty of Pure and Applied Sciences, University of Tsukuba, Ibaraki 305-8577, Japan

**Keywords:** surface degradation, MAPbBr_3_ single crystal, grazing e-beam radiation

## Abstract

To start a step such as some realization of minimized and integrated devices, it requires simply understanding the surface status of hybrid perovskite on the e-beam irradiation because many commercial semiconductor devices are performed with a surface patterning process using e-beam or etching gas. The surface status of CH_3_NH_3_PbBr_3_ (MAPbBr_3_) single crystal was studied after a grazing e-beam irradiation in an ultra-high vacuum. The prepared hybrid perovskite single crystal was irradiated by the 3 degree-grazing e-beam with energy of 15 kV for 10 min using a reflection high-electron energy diffraction technique. The e-beam irradiation on the MAPbBr_3_ hybrid perovskite single crystal induced the deformation from MAPbBr_3_ into MABr, Br_2_, and Pb on the surface. The gas phases of MABr and Br_2_ are depleted from the surface and the Pb element has remained on the surface. As a result of the e-beam irradiation, it formed a polycrystalline-like phase and Pb metal particles on the surface, respectively.

## 1. Introduction

Recently, organic-inorganic hybrid perovskite (OHP) materials have shown impressive results, especially in the case of solar cells, including a power conversion efficiency of over 25%, because of their key physical properties such as high absorption coefficient, high carrier mobility, and long carrier lifetime [1,2,3,4]. However, there are attempts to overcome the critical problems such as material instability and environmental issues caused by Pb-based perovskite, due to which the research on OHP materials is still focused on the solar cell application [5,6,7]. In the case of materials instability, particularly, the degradation of OHP materials under electron beam, UV (ultra-violet), and visible light illumination conditions has been reported [6,7,8,9,10,11]. C. Xiao, et al. had reported the two-steps degradation mechanism such as (1) defect formation caused by irradiation damage and (2) phase transformation induced by electron-beam heating with a high-energy electron beam (5 and 10 kV) [7]. Additionally, the e-beam effect exposed with the perpendicular direction between the e-beam and the hybrid perovskite layer was reported by N. Klein-Kedem, et al. to explain the degradation of the perovskite structure [8]. 

Interestingly, these reports show the behavior on the bulk status in solar-cell device structures because the e-beam was exposed only to the active hybrid perovskite area in a device structure. This approach would be a reasonable to understand the damage effects induced by several variables such as the local heat and radiation damage in the solar-cell application field. However, there is no serious study on the surface status of hybrid perovskite materials by the e-beam irradiation yet. If we try to make a designed pattern on the surface of hybrid perovskite materials using the e-beam irradiation which is a useful and conventional tool in the semiconductor industrials, its effect on the surface should be studied firstly.

In the last decade, researchers have started exploring the possibility of new applications using hybrid perovskite materials such as optoelectronics, memory, laser, THz application, transistor, sensors, and batteries, alongside with specific researches on defects, phonon, and electronic structures, which can be important fundamentals to support future perovskite-applied research [12,13,14,15,16,17,18,19,20,21]. At the same time, we believe that it requires to start a next step such as some realization of minimized and integrated devices needed in the semiconductor industrials [22]. To perform this next step, the etching (or patterning) method and its effect on the surface of active material should be firstly studied. The e-beam is one of candidates for etching (or patterning) and we need to understand the surface status of hybrid perovskite on the e-beam irradiation for etching (or patterning).

In this short communication, we have studied the surface status of the hybrid perovskite single crystal, CH_3_NH_3_PbBr_3_ (MAPbBr_3_), by a grazing e-beam irradiation with high energy. Also, the surface degradation in the hybrid perovskite material is discussed with the remaining Pb particles and the polycrystalline-like phase. 

## 2. Materials and Methods 

**Figure 1:**
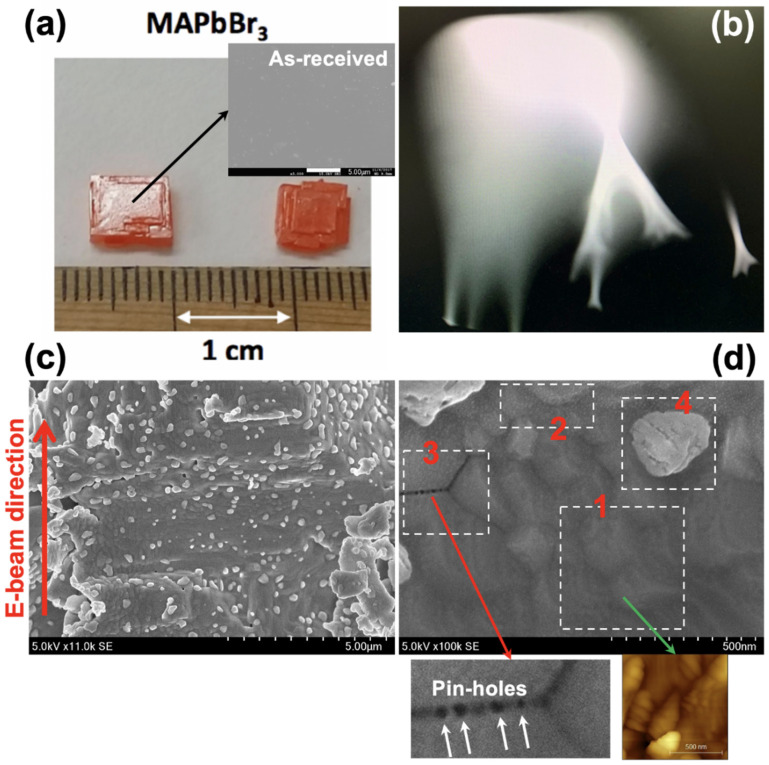
(**a**) The prepared MAPbBr_3_ single crystals with the diameter of 0.6 cm. In the scanning electron microscopy (SEM) measurement for the as-received sample (before the e-beam irradiation), it shows very flat surface. (**b**) The reflection high-energy electron diffraction (RHEED) screen with no spots. It shows a dynamic flowing on the screen. (**c**) The top surface morphology measured by SEM. The recessed surface irradiated by the grazing e-beam and many particles on the surface were observed. (**d**) the four different features (the red numbers) are observed. In the 1 and 3 areas, interestingly, the steps (measured by atomic force microscopy (AFM)) and pin-holes are clearly observed.

MAPbBr_3_ single crystal was grown by an inverse temperature method [23]. A MAPbBr_3_ precursor solution (1.2 M) was prepared by mixing MABr and PbBr_2_ (1:1 molar ratio) in dimethylformamide (DMF). After 30 min of stirring, the solution was filtered through a 0.2 mm pore-size polytetrafluoroethylene (PTFE) syringe filter, and the filtered precursor solution was gradually heated to 90 °C using an oil-bath. Several hundred micron-sized single seed crystals were formed within 1 h. Large-sized (centimeter scale) single crystals were prepared through a crystallization process over 24 h by use of the chosen seed crystals with regular changes of the precursor solution. (Figure 1a)

For a grazing e-beam irradiation on the surface of a single crystal, we used an electron gun in a reflection high-energy electron diffraction (RHEED) system. Before and after loading the sample to the RHEED chamber, we performed the surface scratching using a knife and the annealing process at 100 °C for 10 min, respectively [24]. The base pressure of the RHEED chamber was 1.1 × 10^−9^ Torr. The energy, current, and incident angle were 15 kV, 20 μA, and 3 deg., respectively (Figure 1b). After irradiating for 10 min, we took out the sample and then performed atomic force microscopy (AFM), scanning electron microscopy (SEM), X-ray diffraction (XRD), and high-resolution X-ray photoelectron spectroscopy (XPS). We performed the AFM measurement using SPM-9700 made by Shimazu (Kyoto, Japan). The used SEM system is the HITACHI SU9000 model (Krefeld, Germany) with the acceleration voltage of 5.0 kV and the emission current of 10 μA. The XRD is RINT-TTRIII/NM with Cu*K*_α_ source made by Rigaku (Tokyo, Japan). We used the Versa ProbeII with a monochromated Al*K*_α_ (ULVAC-PHI, Kanagawa, Japan) for all XPS measurements and obtained the C 1*s*, N 1*s*, Pb 4*f*, and I 4*d* core-level spectra. The binding energies were calibrated with reference to the Au 4*f*_7/2_ level (84.0 eV) [25]. 

## 3. Results and Discussion

Interestingly, the dynamic flowing on the screen of RHEED was observed without any atomic patterns or spots [26] (Figure 1b). It might be due to a surface charging effect, and the similar results have been reported before [6,7,8]. 

With the SEM measurement, many small particles and steps have been observed on the surface (Figure 1c) where four patterns on the surface have been shown on the following locations in Figure 1d: location 1) many steps (indicated by the green arrow toward an inset AFM topology image), location 2) large surface roughness, location 3) pinholes/deep boundary (the white arrow), and location 4) particles. (Figure 1d) The e-beam irradiation on the MAPbBr_3_ single crystal seems to cause a polycrystalline-like phase by degradation. It is assumed that these findings can be employed to understanding a degradation mechanism of MAPbBr_3_, processing from location 1 to location 4 in sequence. Firstly, steps and valleys start appearing on the surface. Secondly, the height between steps becomes larger and larger. And then the pinholes occur on the step boundary while forming a grain boundary. Finally, the one grain is isolated and remains a particle. To confirm this assumption, it is required to know its atomic structure and chemical state in bulk and surface, respectively. 

**Figure 2:**
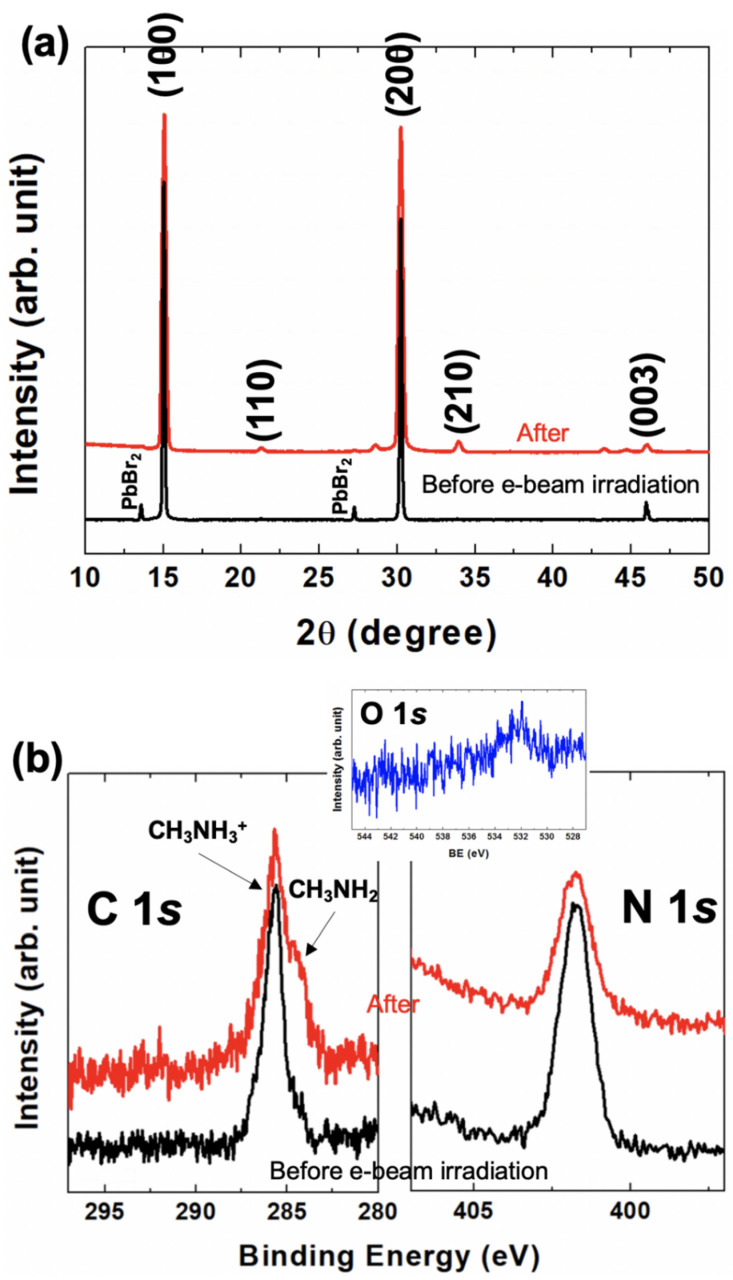
(**a**) The X-ray diffraction (XRD) result before and after the grazing e-beam irradiation. The as-received sample still shows the small PbBr_2_ peak. After the e-beam irradiation, however, it was disappeared. (**b**) The C, N, and O 1*s* core-level spectra before and after the e-beam irradiation. The CH_3_NH_2_ molecular defect has appeared after the e-beam irradiation [28,29]. This defect is due to the formation of a polycrystalline-like phase on the surface [29]. The O 1*s* core-level peak is appeared slightly at around 532 eV which is shown with the physiosorbed oxygen on the surface.

To see the bulk status after the grazing e-beam irradiation, we performed an XRD experiment (Figure 2a). Only the typical MAPbBr_3_ structure was observed and there was no other significant structure [27]. With this result, we confirmed the irradiation effect of the e-beam was only affected on the surface. 

**Figure 3:**
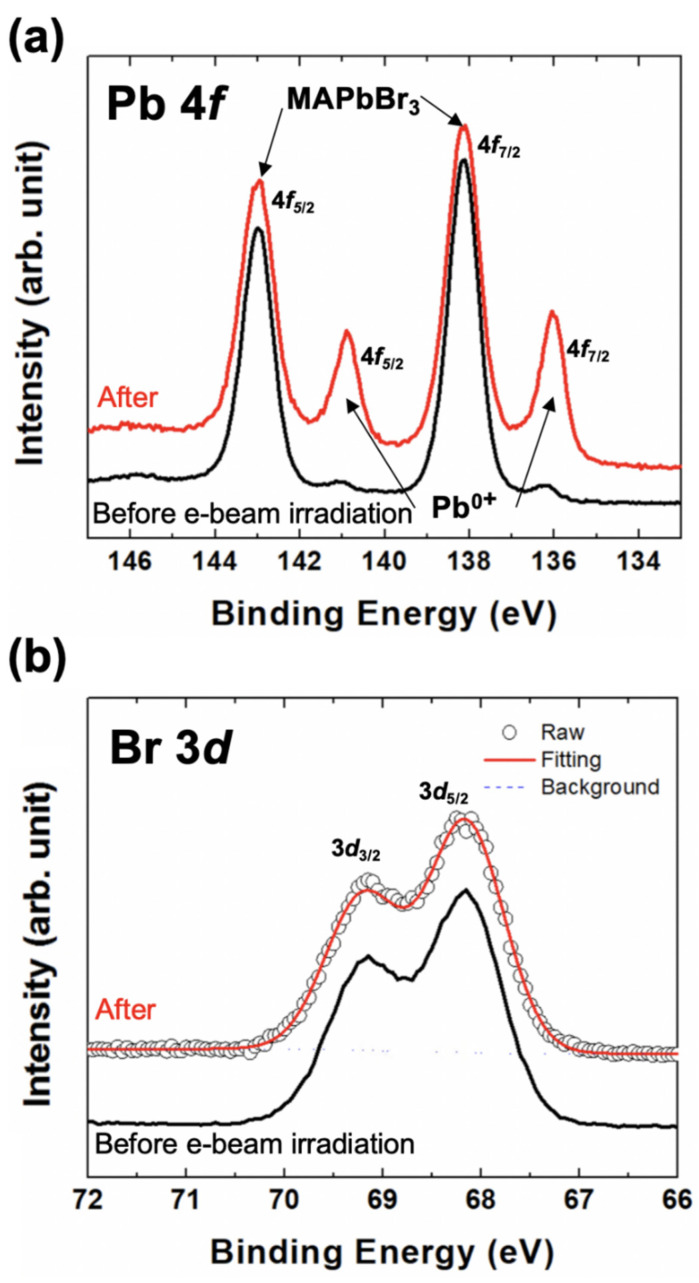
(**a**) The Pb 4*f* core-level spectra before and after the grazing e-beam irradiation. The Pb^0+^ chemical state is clearly observed. (**b**) After the curve fitting, the Br 3*d* core-level spectra before and after the grazing e-beam irradiation shows the single chemical state perfectly.

To see the detailed changes on the surface, XPS was measured with C 1*s*, N 1*s*, Pb 4*f*, and Br 3*d* core-level spectra. (Figure 2b and Figure 3) Also, we measured O 1*s* core-level to confirm the surface contamination after taking out the sample from the RHEED chamber (Figure 2b). The peak with a small trace was observed. If this O 1*s* peak has some chemical bonding with carbon, nitrogen, or other elements, we would observe some different chemical states in C, N 1*s*, Pb 4*f*, or Br 3*d* core-level spectra [25]. However, we could not observe any significant change of chemical states in the C and N 1*s* core-level spectra (Figure 2b). The binding energies of C and N 1*s* core-levels are the same as those of our several previous studies that showed only the hybrid perovskite state [28,29]. It means that oxygen contamination does not make any chemical states bonded to CH_3_NH_3_^+^ cation. Interestingly, we found the Pb^0+^ chemical state (Pb metal) in the Pb 4*f* core-level spectrum (Figure 3a). However, we could not observe any significant chemical state except for the hybrid perovskite state. The single chemical state in the Br 3*d* core-level was confirmed by the curve fitting which was performed with Doniach-Sŭnjić curves, convoluted with a Gaussian distribution function, considering instrumental broadening and background noise due to inelastic scattering was subtracted by the Shirley (integral) method (Figure 3b) [30,31]. 

With these results, we can confirm three important facts—(1) the particles on the surface are pure Pb metal, (2) the surface has no oxide state (which means the surface oxygen is a physical-absorption state), and (3) the surface degradation is occurred by the e-beam irradiation. (Equation (1))

(1) $${\mathrm{MAPbBr}}_{3}\;\left(\mathrm{single}\;\mathrm{crystal}\right)\to \mathrm{MABr}↑+{\;\mathrm{Br}}_{2}↑+\mathrm{Pb}\;\left(\mathrm{on}\;\mathrm{the}\;\mathrm{surface}\right)$$

As a result of grazing e-beam irradiation, the chemical bonding is broken first and then two gas phases, such as MABr and Br_2_, are depleted from the surface. The Pb metal only remains on the surface with the particle structure. The surface becomes the polycrystalline-like phase from the single crystal phase. These understandings are similar to two reports by Z. Dang, et al. and A. Kostopoulou, et al. in all-inorganic halide perovskite materials [10,32]. However, in the case of organic-inorganic hybrid perovskite material, it shows that no remained molecular parts on the surface because of its surface depletion.

## 4. Conclusions

We observed the surface degradation of MAPbBr_3_ single crystal using the 3 degree-grazing e-beam irradiation with the 15-kV high energy. The e-beam irradiation causes the destruction of the chemical structure of MAPbBr_3_ without creating any different chemical states such as etching. However, the Pb metal element with the form of particles remains on the surface. If we use a focused and well-defined sized e-beam, it will be possible to make a designed pattern of an organic-inorganic hybrid perovskite main layer with Pb metal capper or wire on the surface. This experimental idea is suggested for future work.
